# Signet ring cell carcinoma of the rectal stump in a known ulcerative colitis patient

**DOI:** 10.4322/acr.2023.418

**Published:** 2023-01-11

**Authors:** Asmanaz Nadaf, Ibrahim Hassan Al Haddabi, Ramesh Babu Telugu, Mansour S Al Moundhri

**Affiliations:** 1 Sultan Qaboos Comprehensive Cancer Care and Research Centre, Department of Pathology, Laboratory Services, Muscat, Oman; 2 Sultan Qaboos Comprehensive Cancer Care and Research Centre, Department of Medical Oncology, Muscat, Oman

**Keywords:** Colon, Carcinoma, Ulcerative Colitis, surveillance, Rectum

## Abstract

Colorectal carcinoma (CRC) is the third most commonly diagnosed cancer worldwide and is the second most common cause of cancer-related deaths. However, the Omani population shares the major burden as the most prevalent carcinoma. The disease is comparatively higher in males than females. Patients with pre-existing risk factors, including inflammatory bowel disease, are at increased risk of developing neoplasia. Among the various histopathological subtypes of adenocarcinoma in the rectum, signet ring cell carcinoma is the rarest and accounts for approximately 1% of the cases. Given the aggressive nature of this tumor, advanced presentation, stage, and poor prognosis, regular endoscopic surveillance is essential. Hereby, we report a rare case of signet ring cell carcinoma arising in the rectal stump in an already diagnosed and operated patient of Ulcerative colitis.

## INTRODUCTION

Colorectal carcinoma (CRC) is the third most commonly diagnosed cancer worldwide and is the second most common cause of cancer-related deaths.^1^ The incidence of this cancer is higher in younger patients, with the incidence rate increasing by 1.5% in males and 1.6% in females.^2^ Patients with inflammatory bowel disease (IBD) are at increased risk for colorectal cancer. The risk factors include extensive colonic disease, the severity of colonic disease, long disease duration, and the presence of primary sclerosing cholangitis (PSC).^1^

The Omani population witnesses colorectal cancer as the most common cancer. It is the second most common cancer in males and the 4th most in females. The incidence has increased in the last 15 years. The rectum is the most common site of colorectal cancer, followed by the sigmoid colon, ascending colon, descending colon, and transverse colon.^3^ The main risk factors for ulcerative colitis-associated colorectal cancer, include age at onset, duration, the extent of disease, active smoking history, family history of CRC, and concomitant diagnosis of primary sclerosing cholangitis. Patients with risk factors require regular screening and preventing exposure to risk factors.^4^

The pathogenesis of IBD-CRC is related to cellular damage that interacts with tumorigenesis transcription factors leading to dysplasia and adenocarcinoma.^5^ Among the various histopathological subtypes of rectal adenocarcinoma, signet ring cell carcinoma is the rarest and accounts for approximately 1% of all cases. Due to intrinsic tumor biology, this subtype is characterized by aggressive biological behavior and poor prognosis. Signet ring cell carcinoma is commonly seen in young individuals and is associated with unique molecular profiles like microsatellite instability and BRAF mutations.^6^ The present article reports a rare case of signet ring cell carcinoma arising in the rectal stump in an already diagnosed patient with ulcerative colitis.

## CASE REPORT

A 40-year-old gentleman with comorbidities like hypertension, chronic kidney disease, and IBD was referred to our institution for further treatment. The patient had a history of ulcerative colitis with recurrent severe bouts for long-standing for about 13 years and underwent surgery (subtotal colectomy and Ileostomy). The histopathological examination of the colectomy specimen performed in outside hospital was reported as severe pancolitis favoring ulcerative colitis and polyps with low-grade dysplasia. The medical management of ulcerative colitis and its clinical and endoscopic response to the medications used was not documented in our medical records as the patient was referred from outside the hospital.

The possible risk factors for the development of ulcerative colitis-associated colorectal cancer in this patient include long-standing duration of disease for about 13 years and severe pancolitis.

The patient was subjected to surveillance before and after the surgery as per medical records. The patient was on regular follow-up for his fluctuating renal function. The proctoscopy performed in-house showed a rectal stump mass with stricture located at 3 cm from the anal verge, through which the scope couldn’t be passed. Positron Emission tomography (PET) fluorodeoxyglucose (18F-FDG) whole body scan showed increased metabolic activity in the rectum with mildly FDG avid perirectal fat stranding with thickening of mesorectal fascia with mildly FDG avid perirectal, presacral, inferior mesenteric, bilateral external iliac, bilateral internal iliac, bilateral common iliac and aortocaval and paraaortic lymph nodes, likely metastatic.

**Figure 1:**
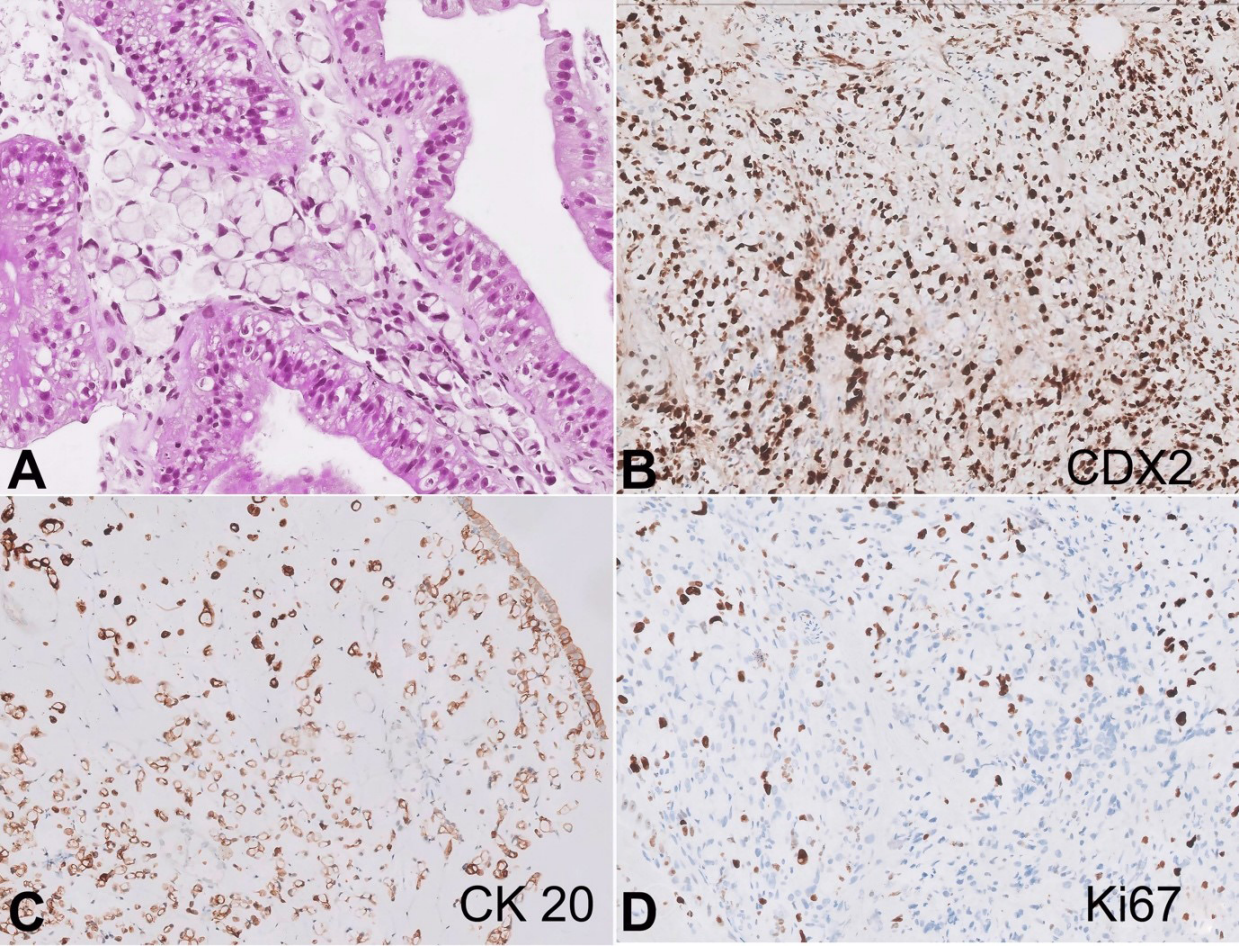
Photomicrographs of the colon. **A -** Signet ring cell carcinoma in colonic mucosa (H&Ex200); **B -** Immunohistochemical positive staining for CDX2 (X200); **C -** CK20 (X200); and D - Ki-67 (X200).

The lesion was biopsied and sent for histopathological examination. The microscopic evaluation showed ulcerated mucosa with reactive epithelial changes and the lamina propria distended with signet ring cells exhibiting typical morphology of peripherally pushed eccentric nuclei and a large mucin vacuole (Figure 1A).

**Figure 2:**
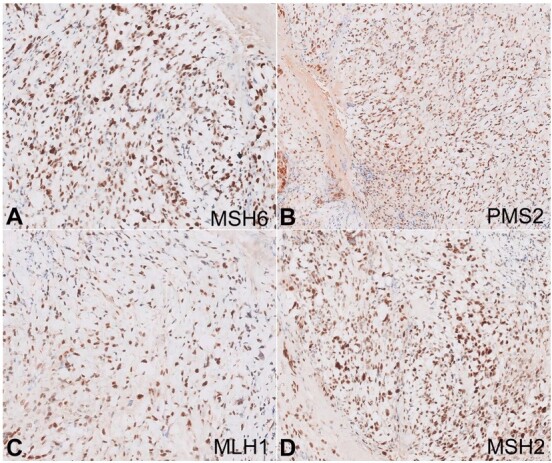
Photomicrographs of the colon. Immunohistochemical reactions positive for: **A -** MSH6 (X200); **B -** PMS2 (X200); **C -** MLH1(X200); and **D -** MSH2 (X200).

Immunohistochemistry shows the tumor cells were positive for CDX-2 (Figure 1B), CK20 (Figure 1C), Cam 5.2, and focally for CK-7. Additionally, the neoplastic cells were strongly positive for p53, and the Ki67 index was 40% (Figure 1D). There was retained nuclear expression for all the Mismatch repair (MMR) proteins (Figure 2A to 2D). The diagnosis of poorly cohesive carcinoma with signet ring cells- MMR proficient was signed out. The molecular profile showed RAS and RAF Wild Type.

The patient did not undergo surgery for the rectal stump due to the fluctuating renal function and to preserve the sphincter function. Hence only biopsy was performed for tissue diagnosis. 6 cycles of mFOLFOX (folinic acid, fluorouracil and oxaliplatin) chemotherapy were administered after the confirmation of adenocarcinoma in the rectal stump biopsy. The follow-up PET scan showed complete resolution of the enlarged nodes and a significant reduction in the FDG rectal uptake. The patient is under follow-up with a good response to chemotherapy.

## DISCUSSION

Compared to the general population, the incidence of colorectal cancer was found to increase among patients with ulcerative colitis.^1^ Many risk factors, such as the long duration of the disease, ongoing inflammation, extensive mucosal involvement, backwash ileitis, and concomitant sclerosis, increase the risk of neoplasia.^4^^,^ 7 The associated dysplasia with ulcerative colitis mostly occurs in the left colon/distal colon, with 44 to 72% of the cases occurring in the recto-sigmoid region.^8^^,^ 9 However, the risk increases with pancolitis compared to left-sided colitis.^1^ Precisely in the present case report, ulcerative pancolitis prevailed for a longer duration of 13 years with the existence of pancolitis.

The pathogenesis of the development of neoplasia in IBD is attributed to repeated cycles of inflammation leading to the initiation and progression of carcinogenesis.^1^ Chronic inflammation often results in re-epithelization of cells, increased cell turnover in colonic mucosa and heightened risk of errors in the cell cycle repair in association with oxidative stress and low internal defense mechanism for detoxification, which has a cumulative effect to promote the progression to neoplasia.^5^ Primarily, changes occurring at the molecular level are TP53 mutations, microsatellite instability, and CPG island hypermethylation. In addition, the variations in the microbiota have also been attributed to the cause of the development of neoplasia in ulcerative colitis.^1^

Adequate surveillance of patients identified as at-risk patients might significantly improve the management of IBD-CRC risk. Current evidence-based guidelines recommend surveillance colonoscopy for patients with colitis 8 to 10 years after diagnosis; further surveillance is decided on the basis of the patient’s risk factors. A new technique, chromoendoscopy, proved to be highly effective for monitoring programs and suggested its use with targeted biopsy.^7^

It is a fact that the risk of neoplasia exists in the rectal stump. However, postoperative endoscopic surveillance could detect dysplasia/cancer at an early stage. Patients with ileorectal anastomosis (IRA) had a greater risk of developing neoplasia than those with ileal pouch-anal anastomosis (IPAA).^9^

In a study done by Belli et al.^10^ it is highlighted that primary signet ring cell carcinoma is (i) an aggressive tumor, (ii) frequently seen in the younger age group at around 40 years, (iii) advanced stages of presentation and (iv) poor prognosis. In the present case, the patient was found to be in a similar age group.

Signet ring cell carcinoma is defined as > 50% of the tumor cells with prominent intracytoplasmic mucin, typically with displacement and molding of the nucleus. This subtype has a very low (1%) incidence rate in the rectum and is more common in the right colon.^8^ In the present case, the origin was on the left side at the ileostomy site in the rectal stump.

The histopathological diagnosis of signet ring cell carcinoma is sometimes challenging due to the presence of benign signet ring cell change. This was first described as a pseudo-neoplastic phenomenon seen commonly in gastric xanthomas and transurethral prostatectomy specimens. However, in the gastrointestinal tract (GIT), most benign signet ring cell changes are reported in areas of injury or ischemia and are usually limited to the mucosa. The morphological features that favor benign signet ring change are circumscription and lobulated appearance, absence of single-cell infiltration and bland cytological features.^11^

In the present case, the distinction from benign signet ring cell change was made based on single-cell infiltration in the lamina propria and cytological atypia. However, this is quite challenging in some cases. The Immuno-markers routinely used to differentiate between benign mimics and true signet ring cell carcinomas are p53 and Ki-67. In a study done by Khan et al.^11^, it was found that the benign signet ring cells were found to be 100% negative for p53 and Ki67. However, in our case, the tumor cells displayed strong positivity for p53 and ki-67 with the labeling index of 40%, indicating malignant signet ring cells. Signet ring cell carcinomas are usually associated with microsatellite instability and BRAF mutations.^6^ In the present case, MMR proteins were intact and proficient, while RAS and RAF showed wild type.

In conclusion, signet ring cell carcinoma is a very aggressive and rare subtype of colorectal adenocarcinoma. Its occurrence at the rectal stump region is rare. Owing to its advanced presentation stage and dismal prognosis, regular endoscopic surveillance must be undertaken in patients with ulcerative colitis, irrespective of surgical status. Further studies are encouraged because there is a lack of literature on the development of neoplasia after colectomy.
